# The direct anterior approach for simultaneous bilateral total hip arthroplasty: a short-term efficacy analysis

**DOI:** 10.1186/s42836-020-00040-w

**Published:** 2020-07-29

**Authors:** Chang Chen, Yiran Yin, Liu Juncai, Ge Chen

**Affiliations:** Department of Orthopaedics, Affiliated Hospital of Southwest Medical University, Luzhou City, Sichuan Province China

**Keywords:** Bilateral total hip arthroplasty, Direct anterior approach, Posterolateral approach, Total hip arthroplasty, Simultaneous THA, One-stage THA

## Abstract

**Purpose:**

Compared to the posterior approach (PA), the direct anterior approach (DAA) can achieve better clinical outcomes for total hip arthroplasty (THA). The purpose of this study was to investigate whether the same advantages associated with the DAA could be attained in patients undergoing simultaneous bilateral THA.

**Method:**

We retrospectively reviewed 89 patients who underwent one-stage bilateral THA through the DAA (group A, *n* = 46) and through the PA (group B, *n* = 43) between June 2015 and November 2017 at our institution. The patients were followed up for a minimum of 1 year. There were no significant differences in gender, age, body mass index (BMI), preoperative hemoglobin level, preoperative Harris hip score (HHS), and preoperative visual analogue scale (VAS) score between the two groups (*P* > 0.05 for all).

**Results:**

The incision length, operation time, intraoperative blood loss, blood transfusion volume, and the length of stay (LOS) were significantly less in group A than in group B (*p* < 0.05). The surgery-related complications were not significantly lower in group A (5.43%) than in group B (10.47%) (*χ*^2^ = 2.209, *p* = 0.112). In 46 cases in group A, one hip had an acetabular anteversion higher than normal value. In both groups, one hip developed aseptic loosening. The HHS was significantly higher in group A than in group B 1, 3, 12 month(s) after operation (*p* < 0.05). The VAS was significantly lower in group A than in group B 1, 3, 12 month(s) after operation. Against the simple Likert scale, comprehensive satisfaction was significantly higher in group A (97.8%, 45/46) than in group B (76.7%, 33/43) (*χ*^2^ = 9.119, *p* = 0.003).

**Conclusion:**

In patients who underwent simultaneous bilateral THA, DAA could significantly relieve pain, accelerate the functional recovery of hip joint and improve the satisfaction more than PA. In clinical practice, however, more attention should be paid to strict compliance to operative indications and the prevention of early complications. The long-term effectiveness warrants further observation.

Total hip arthroplasty (THA) is the most successful and effective intervention for treating degenerative hip diseases [1, 2]. It is estimated that approximately 15 ~ 20% of patients requiring THA are considered for bilateral hip diseases, and satisfactory function could not be entirely regained until both hips received surgical procedures [3, 4].

In recent years, the improvement of surgical techniques and the development of Enhanced Recovery after Surgery (ERAS) pathway allowed simultaneous bilateral THA to be performed more safely and effectively. One-stage procedure could lead to a shorter length of stay (LOS), use of single anesthetic, lower cost, and potentially, an earlier functional recovery [5, 6]. However, whether one-stage procedure could result in increased risks of complications, including venous thromboembolic events (VTEs), higher blood transfusion requirements, and increased need for secondary rehabilitation equipment is still not fully studied.

The direct anterior approach (DAA) for total hip arthroplasty (THA) is shown to be superior to other approaches [7]. This approach has been proven to attain a faster postoperative recovery and to have a lower complication rate compared with other approaches, including the most commonly used posterior approach (PA). Meanwhile, the DAA allowed patients to assume supine position throughout the procedure [8], which might save the operation time and does not require position change during simultaneous bilateral THA.

Though current studies showed that simultaneous bilateral THA could reduce cost in comparison to unilateral THA and may might be associated with a higher rate of complications [9], few studies investigated whether the DAA in B-THA (bilateral total hip arthroplasty) has the same advantages over other approaches. The purpose of this study was to retrospectively compare functional outcomes in patients who underwent one-stage bilateral THA via DAA or through posterolateral approach, performed by a single surgeon.

## Materials and methods

### General information

After approved by the Ethics Committee of our institution, a retrospective review was conducted on the basis of inpatient medical records and pre- and postoperative outpatient clinical charts. From June 2015 to November 2017, 89 consecutive patients who underwent simultaneous bilateral THA in our institution were involved in our study. A senior surgeon performed more than 400 elective primary THAs, in which the DAA was employed in 55.3% of cases and B-THA (bilateral THA) were done in 12.3% of the cases. Forty-six consecutive patients received one-stage B-THA through the DAA, while 43 via the posterolateral approach. All patients gave informed consent.

The inclusion criteria were as follows: (1) bilateral degenerative joint disease of hip, clinically and radiographically diagnosed; (2) body mass index (BMI) ≤ 35; (3) the surgeon believed that patient would benefit from a simultaneous THA procedure despite their pain and disability. Exclusion criteria included: (1) significant shortening of femoral neck or deformities of the proximal femur; (2) severe osteoporosis of the proximal femur; (3) age older than 80 years; (4) Crowe type IV developmental dysplasia of the hip (DDH).

### Types of approach

The senior surgeon performed THA on all patients by using the same surgical technique and peri-operative protocol for both groups. All operations were performed under general anesthesia, and the whole anesthetic procedure was under the management of an experienced anesthetist.

#### The direct anterior approach (DAA)

The patient was positioned in a supine position on a standard orthopaedic table, and the first procedure was performed on more symptomatic side. An iodised incision drape (Ioban 3 M^®^) was applied after conventional draping. The skin incision was made as described by Hüter *et al* [10]. After cutting the skin, the Hüeter interval could be touched and felt by fingers and identified by the fat on the inner edge of the *fascia lata*. The lateral circumflex femoral artery (LCFA) was ligated or coagulated if found to go across the Hüeter interval. The capsule was opened in an L-shaped incision. For the acetabular preparation, three bent retractors were placed separately on the direction of 3, 7 and 12 o’clock of the original acetabulum. The acetabular reaming was performed to maintain an abduction angle of 40 ~ 45° and an anteversion angle of 15 ~ 20°. After the acetabular reaming was satisfactory, cementless acetabular components were implanted into the reamed acetabulum. As for the femoral procedure, the hip joint should be hyper-extended about 30°, and the knee was flexed and placed under the contralateral knee to make it externally rotated and adducted. A hook was used, whenever necessary, to lift the femoral neck if it was difficult to expose the inner wall of the greater trochanter. Then, after full exposure, the reaming maintainer with double eccentricity was used to ream the femur at a moderate anteversion angle (with a combined anteversion angle of 25–35 ° made for males and an angle of 30–45° for females). The reaming from small to large must be meticulously done to prevent the burst fracture of the femur. Fluoroscopy with a C-arm X-ray machine showed that the result was satisfactory, and stem prosthesis and femoral head prosthesis were implanted. After the movement and stability of hip joint were re-tested, an intra-articular suction drain was placed. As aforementioned, the contralateral replacement was performed and care was exercised to make sure the the two legs were of equal length.

#### Posterior approach (PA)

Patients were placed in the lateral decubitus position and the more symptomatic side was operated on at the first procedure. The preparation was the same as for the DAA after conventional draping. The skin incision was made as described by Moore *et al* [11]. Whether to conserve the piriformis tendon was at the discretion of the senior surgeon. The short rotators were sectioned and the joint capsule was opened in a T-shape manner. The soft tissues and osteophytes around the acetabulum was scrupulously cleaned. The acetabulum was reamed at 45 ° abduction angle and 15 ° anteversion angle, and then the prosthesis was implanted. The internal rotation position of the leg was maintained and the femoral prosthesis was implanted after reaming the femur at a 15 ° anteversion angle. Closure of the capsule and reinsertion of the piriformis were achieved by resorbable braided sutures. An intra-articular suction drain was placed. The skin was closed using staples and covered with a non-adherent absorbant dressing reinforced with gauze fixed with a band of Mefix^®^ self-adhesive tape. The patient’s body position was turned, and then the contralateral procedure was performed by using the same method as described above, and, again, care was taken to ascertain that the two legs were of the same length.

### Postoperative management

Once the procedure was finished, patients were transferred into PACU (Postanesthesia care unit). After pain and PONV (postoperative nausea and vomiting) were controlled and active bleeding disappeared, patients were then sent to the ward. Dressing was replaced and drains were removed on POD (postoperative day) 1. Each patient was treated with PCIA (Patient control intravenous anesthesia) pump, in combination with standardized multimodal analgesia: intravenous and oral NSAIDs (celecoxib), tramadol, oxycodone and buprenorphine. If pain remained obvious after oral and intravenous analgesia (VAS score ≥ 5, and the pain affected postoperative functional exercise), an additional nerve block was given. Since the day of operation, a single dose of the cefuroxime (no allergy cases) was injected intravenously and rivaroxaban was given orally for anticoagulation 5 weeks after operation. All patients received local ice dressing from the day of operation.

The key points of the hospital rehabilitation procedure were as follows:

- POD 0: after the effect of anesthesia subsided, the functional exercise should start, including CPM, ankle pump, knee flexion and extension exercise;
- POD 1: The urinary catheter and drainage at surgical sites were removed, and the patients were allowed to stand and walk with crutches or under full load under the guidance of orthopedic doctor and physiotherapist; isometric exercises for femoral and gluteal muscles could begin as well. Then, postoperative X-ray examination and blood tests (including full blood count) were conducted.
- POD 2: Routine wound care was given and compression bandage was used. For exercises, patients were allowed to stand and walk with crutches.
- POD 3 (and the remaining days of hospital stay) Patients engaged in exercising, standing, walking and climbing stairs with crutches. Patients were discharged after the patients were assessed to be able to be discharged safe.

Contact was maintained with all discharged patients via Wechat, a social networking application (Tencent Inc., China) and telephone. They were followed up by a doctor on regular basis.

All patients had three pre-established follow-up appointments with their surgeons:

- 1-month post-op: X-ray examination, evaluation of joint ROMs, planning of new exercises including exercise of muscle strength;
- 3-month post-op: X-ray examination, evaluation of joint ROMs, revision of exercise plan;
- 6-month post-op: new X-ray examination, biomechanical evaluation.

The primary outcome measures included the length of stay (LOS), the total hospitalization cost, and Harris hip score (HHS). And the length of incision, operation time, intraoperative blood loss, transfusion volume and visual VAS were recorded. Patients were followed up regularly 1, 3, 6, 12 month(s) and then annually after the operation, to record the complications, including periprosthetic infection, thromboembolic events, prosthesis dislocation and aseptic loosening.

## Study size and statistical methods

The sample size was calculated according to the primary outcome (the length of stay) using the SAS software package (version 9.0, SAS Institute Inc., Iowa, IA, USA). The calculation was based on a previous study that described the total length of stay of 4.6 days on average (range: from 2 to 17) after one-staged B-THA [5]. We calculated that a minimum of 46 patients (23 in each group) were required for this trial in order to achieve clinically relevant result of an average 3 day decrease in the LOS, assuming a 5% significance level and a 90% power using a two-sided test.

SPSS 21.0 statistical software was employed for statistical analysis. Measurement data were expressed as mean±standard deviation (SD). Repeated analysis of variance was used for comparison between time points before and after surgery in the groups. Paired *t* test was used for pairwise comparison. Independent sample *t* test was utilized for comparison between groups. Comparison of count data between groups was made by using *χ*^2^ test. Inspection level was set at α = 0.05.

## Results

A total of 89 patients were involved, consisting of 29 males and 60 females, with an average age of 60.1 ± 5.4 year-old at the time of surgery. The demographics are shown in Table 1. There existed no significant differences in demographics, including sex (*p* = 1.0), age (*p* = 0.67), BMI (*p* = 0.16), ASA (*p* = 0.74), and preoperative HHS (*p* = 0.11) between the two groups. The preoperative diagnoses included primary osteoarthritis in 58 hips, avascular necrosis in 82 hips and osteoarthritis secondary to DDH in 38 hips.

**Table 1:** Comparison of Patients’ Demographics $\left(\overline{x}\pm s\right)$

Group	*n*	Sex male/female	Age	BMI	ASA	Preoperative HHS
A	46	15/31	59.6 ± 6.0	22.72 ± 3.00	2.0 ± 0.3	41.0 ± 2.6
B	43	14/29	60.2 ± 5.0	21.67 ± 2.90	2.0 ± 0.4	42.0 ± 3.2
Statistic		*p* = 1.000	*t* = −0.500 *p* = 0.620	*t* = 1.609 *p* = 0.115	*t* = − 0.330 *p* = 0.743	*t* = −1.615 *p* = 0.114

The incision length, operation time, total blood loss, transfusion volume, and the length of stay were significantly less in group A than in group B (*p* < 0.05). The patients in group A were followed up for 15–48 months (mean time: 25.3 months) and 12–51 months (mean time: 27.6 months) in group B. The overall incidence of operation-related complications was not significantly lower in group A (6/92, 5.43%) than in group B (9/86, 10.47%) (*χ*^2^ = 2.209, *p* = 0.112). One hip (1.08%) in group A had a acetabular anteversion higher than normal value. Aseptic loosening occurred in 1 hip in either group respectively and the two patients both underwent revision surgeries. The HHS and VAS scores at each time of follow-up after operation were significantly improved in both groups when compared with preoperative scores (*p* < 0.05). The Harris score 1 and 3 month(s) after operation and the VAS score 3 days after operation in group A were significantly better than those of group B (*p* < 0.05), but no significant difference was found between the 2 groups at the last follow-up (*p* > 0.05). Analysis using the simple Likert scale showed that comprehensive satisfaction was significantly higher in group A (97.8%, 45/46) than in group B (76.7%, 33/43) (*χ*^2^ = 9.119, *p* = 0.003).

In group A, 4 hips (4.34%) suffered from skin numbness, which might be ascribed to the injury of the LFCN, and all of them recovered spontaneously after 3 months without special treatment. In group B, 1 hip (1.16%) had the contusion of the sciatic nerve, which recovered after treatment. In group A, 1 hip (1.08%) developed intraoperative trochanteric fracture, which was bound with double-strand steel wires and healed 3 months postoperatively. In group B, 2 hips (2.32%) had a proximal femoral fracture, which was bound with steel wire and then healed. Two hips (2.32%) in group B suffered from a dislocation due to the position change during the operation, and it was reduced successfully under anesthesia. Two hips (2.32%) in group B developed posterior tibial vein thrombosis and recovered after regular anti-coagulation treatment. No infection, heterotopic ossification, pulmonary embolism and other complications occurred in either group. In addition, in group B, 3 patients had the leg-length difference of more than 1 cm, and in 2 patients, their legs were not anatomically restored to the length of the other leg, and in 1 patient, one leg was anatomically restored to the length of the other leg. In total, 5 hips (5.81%) were abnormal. The data of the two groups are detailed in Tables 2, 3 and 4.

**Table 2:** Comparison of Harris scores at pre- and post-operation between 2 groups $\left(\overline{x}\pm s\right)$

Group	n	Preoperative	One month after operation	Three months after operation	Last follow-up
A	46	41.0 ± 2.6	61.8 ± 4.4	84.1 ± 6.8	90.9 ± 5.3
B	43	42.0 ± 3.2	57.4 ± 6.3	80.9 ± 5.9	89.6 ± 5.2
Statistic evaluation		*t* = −1.615 *p* = 0.114	*t* = −3.586 *p* = 0.01	*t* = − 2.148 *p* = 0.037	*t* = − 1.112 *p* = 0.273

**Table 3:** Comparison of VAS scores at pre- and post-operation between 2 groups $\left(\overline{x}\pm s\right)$

Group	n	Preoperative	Three days after operation	Last follow-up
A	46	6.5 ± 1.7	2.6 ± 0.9	1.1 ± 0.9
B	43	6.1 ± 1.5	3.3 ± 1.4	1.4 ± 1.2
Statistic evaluation		*t* = − 1.294 *p* = 0.203	*t* = − 2.960 *p* = 0.005	*t* = −1.355 *p* = 0.183

**Table 4:** Comparison of clinical indexes between 2 groups $\left(\overline{x}\pm s\right)$

Group	*n*	Incision length (cm)	Operation time (min)	Intraoperative blood loss (ml)	Transfusion volume (ml)	Hospitalization time (d)
A	46	7.7 ± 0.9	216.8 ± 19.9	322.6 ± 27.0	322.5 ± 157.9	5.3 ± 1.2
B	43	11.0 ± 1.1	255.8 ± 23.6	389.5 ± 26.6	418.0 ± 70.0	8.5 ± 2.3
Statistic evaluation		*t* = −17.216 *p* <0.001	*t* = −9.262 *p* < 0.001	*t* = − 11.782 *p* < 0.001	*t* = −6.374 *p* < 0.001	*t* = − 9.636 *p* < 0.001

**Fig. 1:**
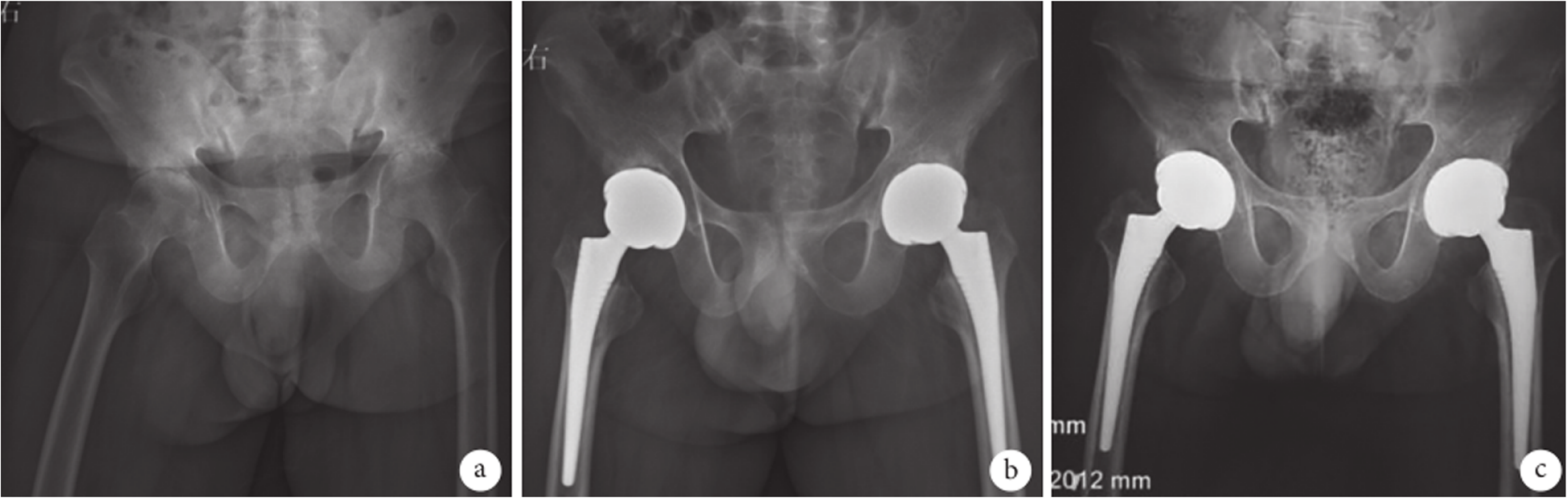
X-ray films of a 35-year-old male patient with ankylosing spondylitis with double-hip osteoarthritis in group A. **a**. Before operation; **b**. 3 months after operation; **c**. 18 months after operation

**Fig. 2:**
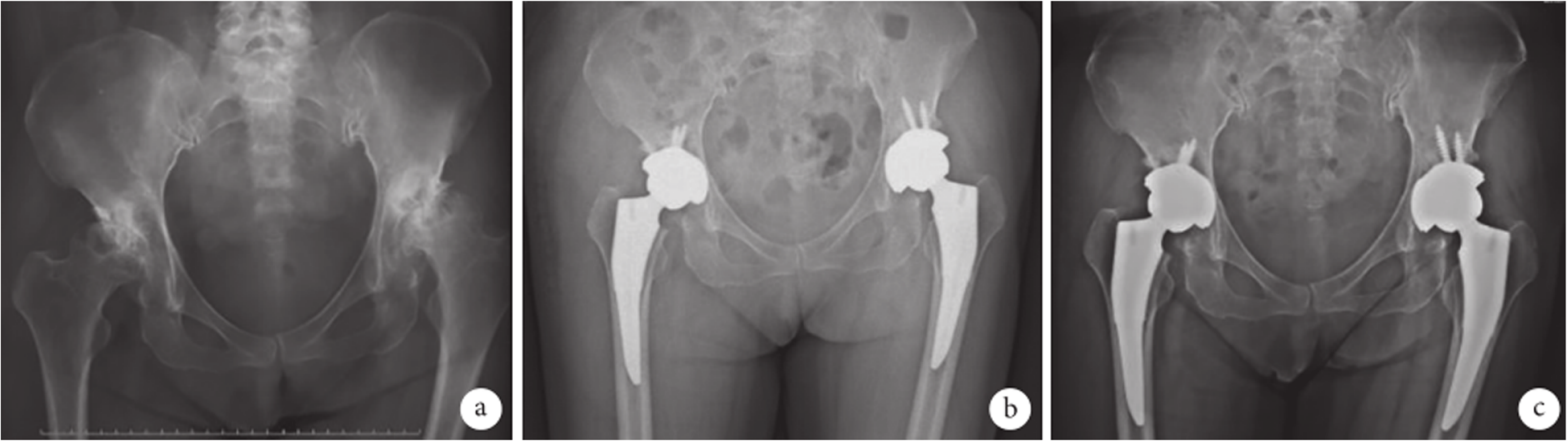
X-ray films of a 62-year-old female patient with bilateral congenital hip dysplasia and secondary bilateral osteonecrosis of femoral heads in group B. **a**. Before operation; **b**. 3 months after operation; **c**. 18 months after operation

The results of radiological evaluation were as follows. First, the acetabular anteversion angle in group A was 16–24 °, with an average of 19 °, and in 1 hip (1.08%), the angle was 24°, which exceeded the normal range (15–20 °) and the abduction angle was 40 ~ 43°, with an average of 42°, all being within the normal range (40 to 45 °). In group B, the acetabular anteversion angle was 15 to 19°, with an average of 16°, and the abduction angle was 41 to 45°, with an average of 44 °, all being within the normal range. Second, analysis using the Engh method exhibited that, 3 months after the operation, 1 hip (1.08%) in group A was found to have extensive sclerotic band surrounding the inner side of the femoral stem prosthesis. And a translucent band was revealed around the prosthesis and there were signs of subsidence. This unstable fixation might result from the use of undersized prosthesis. In group B, 1 hip (1.16%) was found to have a broad sclerotic band around the femoral stem prosthesis 12 months postoperatively, with a translucent band > 1 mm. The stem was unstable and might be attributed to the initial stability of the prosthesis. There was no obvious bone resorption in the rest of the femoral prostheses in the two groups. The proximal end of the stem was tightly connected to the bone cortex and the distal ends were well-matched with the medullary cavity (See Figs. 1, 2).

## Discussion

Simultaneous bilateral THA might pose potential risks, including those associated with longer operative/anesthesia time, more blood loss, higher likelihood of pulmonary embolism. Therefore, some surgeons might refrain from performing simultaneous bilateral THA, especially, since the safety of one-stage bilateral THA has not definitively been proven to be equivalent to that of staged THA [12]. The blood loss in THA is mainly caused by the exudation of femoral medullary cavity and acetabular surface. Prolonged operation time and huge trauma resulting from bilateral THA may lead to more blood loss and transfusion, which were the risk factors for the infection and deep vein thrombosis (DVT) in lower limbs [13]. However, a study of 160 cases showed that simultaneous B-THA did not lead to higher complication or blood transfusion rates compared to staged THA. And in cases of bilateral disease, one-stage B-THA can significantly decrease the total length of stay and, thus, hospitalization costs [14]. Recently, because of introduction of advanced surgical and anesthesia technologies, many studies seemed to support the notion that simultaneous bilateral THA might be a better option, especially for younger patients who are concerned about absence from work.

The application of the DAA in one-stage bilateral THA was rarely reported. Brown *et al* [15] suggested that the DAA could be safely and effectively applied in one-stage bilateral THA without resulting in increased complications compared to sequential hip arthroplasty if perioperative pain and blood loss could be properly managed. In a retrospective review covering 325 consecutive cases, Tamaki *et al* [16] concluded that, due to supine position and the minimally invasive nature of the DAA, the rate of systemic complications such as dislocation was lower with one-stage bilateral THA. We believe that the DAA can facilitate the bilateral procedure because this approach does not need position change. On the other hand, the traditional posterolateral surgical approach requires patients to assume a lateral decubitus position and thus entails repositioning and increases the risk of contamination, wound dehiscence and dislocation. And our results also indicated that the DAA might have several advantages: Supine position facilitated the anesthesia management and the comparison of the length of the lower limbs during the operation. It accelerated the patient’s recovery, shortens the length of stay, and eventually save the total cost. Simultaneous operation could avoid the impact of the functional exercise imposed by the other side after unilateral replacement. A study of 448 cases [17] suggested that B-THA does not lead to significant delay in overall recovery or functional recovery, associated with one-stage B-THA through the DAA. In our study, the LOS was significantly shorter in group A than in group B. Our study also suggested that those elderly patients and those with American Society of Anesthesiologists (ASA) scores of 3 or 4 should be carefully selected against indications. Meanwhile, the postoperative complications were significantly less in group A than in group B. One patient in group B diagnosed as having ankylosing spondylitis developed fracture of proximal femur during surgery. This might result from the osteoporosis caused by the long-time intake of glucocorticoids. And the fracture healed after being tied up with steel wire. Two hips had dislocation in group B (on day13 & day 26). They were successfully managed by closed reduction. Meanwhile, our study also showed that there were no significant differences in operation time, total blood loss and the LOS. We believe that reducing the surgical trauma and total blood loss is important for lowering complications of simultaneous bilateral THA. Therefore, patient selection in strict accordance with indications, use of minimally invasive surgical technology in combination with the application of ERAS in perioperative management could ensure the safety and efficacy of simultaneous bilateral THA.

The concept of ERAS, or fast-track surgery, was first introduced in 1997 by Dr. Henrik Kehlet [18], and involves evidence-based perioperative optimization with multidisciplinary approaches to reduce operative stress and expedite postoperative recovery. Orthopaedically, the advantages include shorter hospital stay and lower postoperative morbidity and mortality [19]. The key to the achievement of the ERAS is minimally invasive operation. Yoshii *et al* [20] showed that THA was associated with reduced length of stay and low complication rates when performed in a fast-track setting. The direct anterior approach for THA was characterized by its true internervous and intermuscular surgical approach which can lead to faster recovery, quicker functional return, and less pain. Tsiridis *et al* [21] compared the safety and efficacy of bilateral THA in the same period through meta-analysis of homogeneous data, and showed that there were no statistically significant differences in thrombosis and dislocation when comparing staged or unilateral procedures with bilateral simultaneous THA. And higher blood transfusion requirements were expected following bilateral simultaneous THA than after staged or unilateral THA. Meanwhile, Parvizi *et al* [22] retrospectively reviewed records of 319 patients who underwent one-stage bilateral THA, and found that, compared to the lateral approach, calculated blood loss and blood transfusion were significantly lower in the DAA group. In our opinion, the procedure is safe and feasible when the indications are strictly followed and the minimally invasive technology is used. Moreover, the ERAS pathway, aimed to strengthen the perioperative management, can make simultaneous bilateral THA even safer and more effective, and might outperform staged replacement in terms of safety and efficacy.

Our study suggested that the following points should be considered before using DAA for simultaneous bilateral THA. First, patients’ BMI should be less than or equal to 35. York *et al* [10] found that in the DAA surgery, obese patients (BMI ≥ 35) had higher infection and revision rates. Hartford *et al* [23] also found that obese patients with a BMI ≥ 40 were at higher risk of postoperative femoral fracture. Second, patients with short neck deformity, femoral bone marrow stenosis or abnormal morphology, severe osteoporosis at the proximal femur and Crowe type IV DDH should not be selected for this approach.

Despite those advantages, attention should be paid to the early complications resulting from DAA. First, injury of lateral femoral cutaneous nerve (LFCN) is possible. Four hips in group A were found to have LFCN injury. All of them recovered in 3 months after operation. Ozaki *et al* [24] believed that smaller femoral offset was a significant risk factor for LFCN injury following THA through the DAA. And their recommendations are that care should be exercised during the skin-fascia incision and subcutaneous exposure and that excessive retraction of the sartorius and the *fascia lata* should be avoided. Moreover, a normal femoral offset could also effectively protect LFCN. Second, intraoperative fractures, including trochanteric fractures and femoral shaft fractures, should be addressed. In this study, trochanteric fracture occurred in 1 hip in group A, and the bone healed after fixation with double-stranded steel wire ring. Therefore, during the femoral procedure, the soft tissue of the proximal femur and the joint capsule must be fully released, and the proximal femur should not be recklessly lifted. Third, muscle pressing injuries, especially the lateral squeezing of the *fascia lata* should be avoided.

Radiological evaluation yielded several findings. First, larger anteversion angle of the acetabular cup might result from DAA. The study of Chen *et al* [25] showed that cup anteversion was, on average, 3° higher in the DAA compared to the direct lateral approach, and there was no significant difference in abduction angle between them. Second, femoral prosthesis is relatively easy to choose. Rivera *et al* [26] suggested that, due to the technical difficulty of femoral preparation and the concern of possible related complications, undersized stems might be implanted more frequently through DAA than through PA, especially if intraoperative imaging was not used. In our study, although intraoperative fluoroscopy was used, 1 hip femoral prosthesis still developed subsidence (3 months after operation) and the acetabular anteversion angle in 1 hip was large in group A. And their abduction angles were all normal.

This study had some limitations. First, due to certain inherent features, the retrospective design is subject to some biases. The researchers, outcome assessors and patients were not blind to treatment group. Second, the cohort distribution was mainly chosen by the surgeon, and this might result in some selection biases. Additionally, the sample size of the study was relatively small and both groups had very low complication rates, which rendered it hard to identify the association of the procedure with complications. These limitations could be avoided in future larger and long-term follow-up studies. The weakness of retrospective review could be effectively overcome by well-designed prospective randomized controlled trials.

## Conclusion

Overall, our results suggest that, the application of DAA in minimally invasive one-stage bilateral total hip arthroplasty could significantly relieve pain, promote the recovery of hip joint function, and improve the patient satisfaction compared to the traditional posterolateral approach. But in clinical practice, more attention should be paid to strict compliance with the indications and prevention of the early complications. The long-term efficacy needs to be further observed.

## Data Availability

The datasets used and analyzed during the current study are available from the corresponding author on reasonable request.

## Abbreviations

THA: Total hip arthroplasty
DAA: Direct anterior approach
PA: Posterolateral approach
HHS: Harris hip score
VAS: Visual analogue score
LCFA: Lateral circumflex femoral artery
PACU: Postanesthesia care unit
PONV: Postoperative nausea and vomiting
POD: Postoperative day
PCIA: Patient control intravenous anesthesia
